# Systematic Review: HIV, Aging, and Housing—A North American Perspective, 2012–2023

**DOI:** 10.3390/healthcare12100992

**Published:** 2024-05-11

**Authors:** Arthur S. Chaminuka, Gayle Prybutok, Victor R. Prybutok, William D. Senn

**Affiliations:** 1 Department of Rehabilitation and Health Services, University of North Texas, Denton, TX 76205, USA; gayle.prybutok@unt.edu; 2 G. Brint Ryan College of Business and Toulouse Graduate School, University of North Texas, Denton, TX 76205, USA; 3 Dr. Sam Pack College of Business, Tarleton State University, Stephenville, TX 76401, USA; wsenn@tarleton.edu

**Keywords:** HIV, older adults, stigma, sexuality, housing, homelessness

## Abstract

Advances in anti-retroviral therapy (ART) have decreased mortality rates and subsequently led to a rise in the number of HIV-positive people living longer. The housing experiences of this new population of interest—older adults (50 years and older) living with HIV—are under-researched. Understanding the housing experiences and unmet needs of older people with HIV can better provide comprehensive care services for them. This study’s systematic review evaluated the peer-reviewed literature reporting housing access/insecurity/assistance/options, housing impact, and unmet needs of older individuals living with HIV in North America from 2012 to 2023. Furthermore, Latent Semantic Analysis (LSA), a text-mining technique, and Singular Value Decomposition (SVD) for text clustering were utilized to examine unstructured data from the abstracts selected from the review. The goal was to allow for a better understanding of the relationships between terms in the articles and the identification of emerging public health key themes affecting older adults living with HIV. The results of text clustering yielded two clusters focusing on (1) improvements to housing and healthcare services access and policies and (2) unmet needs—social support, mental health, finance, food, and sexuality insecurities. Topic modeling demonstrated four topics, which we themed to represent (1) a holistic care approach; (2) insecurities—food, financial, sexuality, and other basic needs; (3) access to housing and treatment/care; and (4) homelessness and HIV-related health outcomes. Stable housing, food, and healthcare services access and availability are critical elements to incorporating comprehensive, holistic healthcare for older adults living with HIV. The aging population requires high-priority policies for accessible and equitable healthcare. Clinicians and policymakers should address individual barriers, adopt a patient-centered approach, increase doctor visits, provide competency training, ensure long-term follow-up, involve families, and improve patient education in care management, contributing to HIV/AIDS geriatric care models.

## 1. Introduction

Access to, availability of, and affordability of adequate housing are fundamental human rights, according to the United Nations (U.N.) [1]. According to the U.N.‘s Universal Declaration of Human Rights, adequate housing includes more than essential shelter [1]. The main structural factor affecting HIV vulnerability, risk, and health effects is housing status. When placed strategically, housing serves as a transitional structural element, connecting systemic social, cultural, and economic exclusion processes to the more concrete physical and social environments in which individuals reside and are employed [2]. Housing issues have emerged due to the HIV epidemic; approximately half of the 1.2 million HIV-positive individuals require housing assistance [3]. To represent the current HIV epidemic trend, highly active antiretroviral therapy (HAART) has helped HIV-positive individuals live longer. Approximately 50% of the 1.3 million people living with HIV/AIDS (PLWHA) in the U.S. are 50 years and older; by 2030, this age group is projected to be 70% [4]. In 2021, 36,136 people were diagnosed with HIV in the U.S. [5], and of these newly diagnosed patients, about one in six are 50 years of age or older [6]. This subpopulation is experiencing rapid growth in North America due to improved and highly active antiretroviral medication. New caregiving challenges are emerging, coupled with homelessness/home insecurity and cumulative disadvantage issues encountered during their life course. For persons living with HIV/AIDS (PLWHA), housing is a unique and essential health determinant. It has wider manifestations and systemic processes of marginalization and inequality, which are factors that increase HIV vulnerability and lead to generally poor outcomes. An increased occurrence of comorbidity and multimorbidity levels has been documented in recent studies in older adults with HIV relative to the same age group without HIV, attributed to a significant research gap in comprehending the effects of HIV infection and ART (Anti-retroviral therapy) on the biological aging process [7,8]. The overall cost of HIV management in this subpopulation will continue to increase as life expectancy rises and the structure of the costs shifts from ART-induced comorbidities, therapy, and adverse events that are emerging [9]. The intersection of double, or in some cases, multiple stigmas (negative social phenomena) due to ageism, HIV, sexuality, or racism has not only physical implications but also psychological and social changes and inadequate involvement with the medical system [10,11,12,13] and contributes immensely to the health-related quality of life. The 50-year-old cut-off point for “older adult with HIV” is based on diminished physical function, increased frailty, and other geriatric symptoms that are more common than in the general population [13] versus the commonly used cut-off of age 65 and over.

The housing transitions experienced by older adults vary from assisted living, retirement homes, subsidized housing, short- and long-term care facilities, and owned or rental houses. In this article, we seek to recognize the formal and informal support networks currently available to house and address the complex needs of older people living with HIV. The housing experiences of older adults living with HIV are under-researched. A search of the literature [14] examined articles from 1990 to 2012 on housing challenges encountered by older adults with HIV in acquiring safe and appropriate housing, yielded only one article [15], a qualitative study examining the housing experiences of older adults with HIV. This systematic PRISMA review explores the literature from 2012 to date on challenges, lived experiences, homelessness, housing access/insecurity/assistance/options, housing impact, and unmet needs of older adults living with HIV in North America. The review approach is a credible, transparent, reliable, and repeatable methodology. Its application enhances healthcare delivery systems’ use of evidence-based care management and policymaking to address emerging public health issues. In addition, a text-mining approach was applied, Latent Semantic Analysis (LSA), to analyze unstructured data from the abstracts selected in the PRISMA review to better understand the relationships between terms in the articles, noting the emerging key topics/themes concerning public health issues affecting older adults with HIV. The housing problems that older adults with HIV encounter will be a crucial area of emphasis, highlighting the significance of housing as a social determinant of health and public health intervention. The housing situation of older HIV-positive adults is poorly understood and under-researched. Due to the effectiveness of anti-retroviral therapy (ART) and a rise in the number of older adults contracting HIV, this subpopulation is growing. The results will inform future housing policies and initiatives involving PLWHA, with a focus on older or aging adults living with HIV/AIDS.

## 2. Research Questions

The research questions will explore insights and themes that support knowledge synthesis from PRISMA to reflect the current evidence on social determinants of health and unfulfilled needs for older PLWHA. The objectives of this study are to understand and address the housing experiences, options, and unmet needs of older HIV-positive individuals, thereby providing informed recommendations for comprehensive care services to enhance their well-being and quality of life. The research questions guiding this study include the following: How does stigma affect fair housing options (retirement/assisted living/other forms of housing)? How does sexuality correlate with housing access and maintenance? How important is housing as a health determinant for older PLWHA? Is HIV/AIDS stigma and discrimination still an issue for older PLWHA? This extensive PRISMA systematic review investigates the current knowledge of homelessness, housing access/insecurity/assistance/options, housing implications, and unmet needs of older PLWHA in North America.

## 3. Methodology

**Figure 1:**
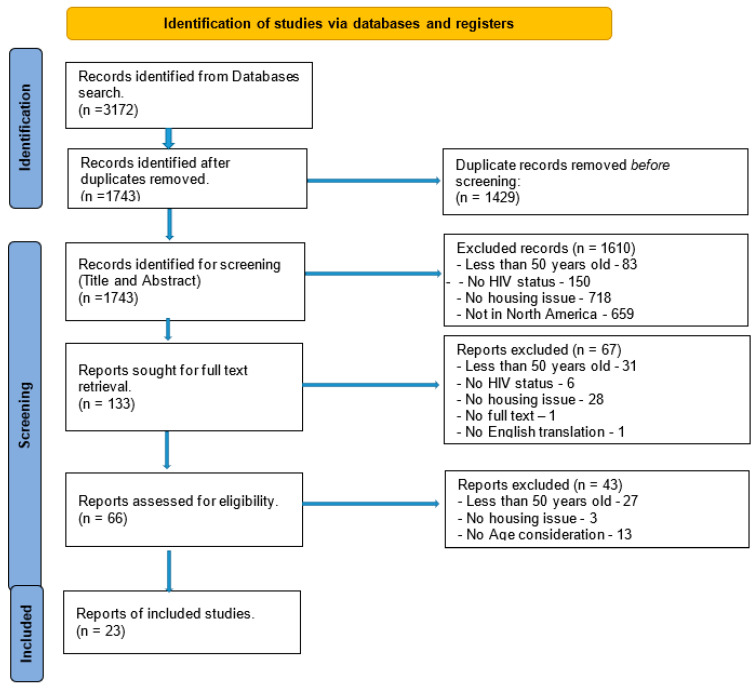
PRISMA Systematic Review flowchart.

This study used a systematic review (PRISMA) to provide a detailed, transparent investigation of previous research on the current knowledge of homelessness, housing access/insecurity/assistance/options, and the implications of housing on older PLWHA in North America (see Supplementary Materials). The process was documented as illustrated in the flow chart (Figure 1) to maintain transparency, integrity, and credibility of the PRISMA systematic review [16].

## 4. Literature Search and Selection Criteria

A search was conducted using EBSCO Host databases, which retrieved most articles from MEDLINE, Academic Search Complete, APA PsycINFO, Health Source: Nursing/Academic Edition, and Psychology and Behavioral Sciences Collection. EBSCO’s databases provide a robust and comprehensive collection of the latest evidence-based and accurate medical literature resources in biomedical, nursing, and allied health that enhance healthcare practitioners’ research experiences. The Boolean phrase search criteria included the terms HIV or aids or acquired human immunodeficiency syndrome or human immunodeficiency virus AND elderly or aged or older or elder or geriatric AND housing or (retirement home) or (nursing homes) or (care homes) or (long-term care) or (residential care) or (aged care facility).

Systematic Literature Review Search Terms: Inclusion and Exclusion Criteria.

**Search Inclusion and Exclusion Criteria**Search words/termsHIV or AIDS or acquired human immunodeficiency syndrome or human immunodeficiency virus AND  elderly or aged or older or elder or geriatric AND housing or (retirement home) or (nursing homes) or (care homes) or (long-term care) or (residential care) or (aged care facility).Inclusion CriteriaDate of publication—January 2012–March 2023 (11 years) HIV diagnosis Language of publication—English, translation to English availability Type of articles includes full text/scholarly and peer-reviewed Study methodology—quantitative, qualitative (interviews, focus groups, ethnography), mixed-methods Geographic location(s) Country—North America (USA and Canada) Age of subjects > 50 yrsExclusion CriteriaAge of subjects < 50 yrs HIV status—negative Country—Not USA or Canada Abstract with an unavailable full article Language—not English/Translation not available Date of publication—(>10 yrs) Type of publication—letters, editorials, non-peer-reviewed articles, dissertations/theses, news and conference articlesLocationUSA and Canada

## 5. Results

A total of 3172 articles were retrieved using the defined selection criteria (search term, inclusion, and exclusion criteria). Duplicate records, 1429 articles, were removed from screening since the search was extensive and occurred in several databases. It is natural to have duplicate records, which can occur in other stages of the systematic review process. The remaining 1743 articles were identified for title and abstract screening. A total of 1610 articles were excluded because they did not meet the inclusion criteria. Full texts were retrieved for the 133 remaining articles for further screening/review, and 67 articles failed to meet the inclusion criteria. A total of 66 articles remained for in-depth review for eligibility. In this review, a further 27 articles were excluded for not meeting the age threshold, three did not address any housing issues, and 13 had no age consideration, addressing people living with HIV in general. The final selection of articles for in-depth synthesis was 23. Summary of exclusion reasons: duplicates (1429), less than 50 years old (141), no HIV diagnosis (156), no housing issue (749), not in North America (659), no age consideration (13), no full text (1), and no English translation (1).
**Systematic Review Characteristics of Included Studies: Summary****Study Design****Country****Author(s)****Date****Characteristics**Furlotte et al., 2012 [15]Mar-12Aging/Elderly housing options, Stigma (HIV-related, sexuality, race), Sexuality, other—poverty, racequalitativeCanada, OttawaLane et al., 2013 [14]Jan-13Aging/Elderly housing options, Stigma (HIV-related, sexuality, race), Housing-related HIV health outcomes/healthcare utilization, other—Nurse caregiver impact, HIV/AIDS staff education systematic lit. reviewNorth AmericaSolomon et al., 2014 [17]Feb-14Aging/Elderly housing options, Stigma (HIV-related, sexuality, race), Housing-related HIV health outcomes/healthcare utilization, other—poverty, food and financial insecurityqualitativeCanada, OntarioArnold et al., 2017 [18]Jan-17Aging/Elderly housing options, Stigma (HIV-related, sexuality, race), Housing-related HIV health outcomes/healthcare utilization, Sexuality, Social and community support, Other—poverty, food and financial insecurityqualitativeUSA, San FranciscoCox and Brennan-Ing, 2017 [19]Jan-17Aging/Elderly housing options, Stigma (HIV-related, sexuality, race), Housing-related HIV health outcomes/healthcare utilization, Sexuality, Social and community support, Other—case management, clinical provider referrals, mental health, and substance use treatment, housing assistance, legal services, nutrition, transportation, home carereview/commentaryUSASiou et al., 2017 [20]May-17Aging/Elderly housing options, Stigma (HIV-related, sexuality, race), other—basic HIV transmission education and awareness for LTC staffqualitative and quantitativeCanada, TorontoTobin et al., 2018 [21]Jan-18Aging/Elderly housing options, Stigma (HIV-related, sexuality, race), Housing-related HIV health outcomes/healthcare utilization, Sexuality, Social and community support, Other—financial insecurity, physical health, mental health, relationships with family (safe physical space for socializing and collaborating with their peers (sexual minority populations))quantitativeUSA, BaltimoreSolomon et al., 2018 [22]Apr-18Aging/Elderly housing options, Stigma (HIV-related, sexuality, race), Social and community support, Other—fulfilling gendered and family roles. Financial insecurityqualitativeCanadaSok et al., 2018 [23]May-18Aging/Elderly housing options, Housing-related HIV health outcomes/healthcare utilization, other—role cultural differences may play in long-term caregiving preferences and advance planning care. (food, clothing)quantitativeCanada, OntarioNguyen et al., 2019 [24]Feb-19Aging/Elderly housing options, Stigma (HIV-related, sexuality, race), Housing-related HIV health outcomes/healthcare utilization, Sexuality, Social and community supportquantitativeUSA, Los Angeles & New OrleansBaguso et al., 2019 [25]Apr-19Aging/Elderly housing options, Stigma (HIV-related, sexuality, race), Housing-related HIV health outcomes/healthcare utilization, Sexuality, other trans-gender hormonal usequantitativeUSA, San FranciscoOlivieri-Mui, 2019 [26]May-19Aging/Elderly housing options, Housing-related HIV health outcomes/healthcare utilization, Social and community support, other—financial insecuritycommentaryUSAJustice and Akgün, 2019 [27]Jul-19Aging/Elderly housing options, Stigma (HIV-related, sexuality, race), Housing-related HIV health outcomes/healthcare utilizationcommentaryUSAWhittle et al., 2020 [28]Jan-20Aging/Elderly housing options, Stigma (HIV-related, sexuality, race), Housing-related HIV health outcomes/healthcare utilization, Sexuality, other financial insecurity, physical and mental healthqualitativeUSAWainwright et al., 2020 [29]Jun-20Aging/Elderly housing options, Housing-related HIV health outcomes/healthcare utilization, quantitativeUSA, DCYoo-Jeong et al., 2020 [30]Jul-20Aging/Elderly housing options, Stigma (HIV-related, sexuality, race), Housing-related HIV health outcomes/healthcare utilization, Social and community support, other—trauma, financial insecurities, loneliness, social exclusionquantitativeUSA, Georgia, ATLChayama et al., 2020 [31]Aug-20Aging/Elderly housing options, Stigma (HIV-related, sexuality, race), Housing-related HIV health outcomes/healthcare utilization, other—Drug abusecommentaryUSAKoehn et al., 2021 [32]Jan-21Aging/Elderly housing options, Stigma (HIV-related, sexuality, race), Housing-related HIV health outcomes/healthcare utilization, Sexuality, Social and community support, Other—finacial insecurityquantitativeCanadaCherry et al., 2021 [33]Jun-21Aging/Elderly housing options, Stigma (HIV-related, sexuality, race), Housing-related HIV health outcomes/healthcare utilization, Sexuality, Social and community support, Other—financial insecurity, provider competency, isolation/lonelinessqualitativeUSA, Los AngelesMurzin et al., 2022 [34]Sep-22Aging/Elderly housing options, Stigma (HIV-related, sexuality, race), Housing-related HIV health outcomes/healthcare utilization, Sexuality, Social and community support, Other—Trauma, uncertainty, and alternative is to access support to age in place, at home, but this option is also fraught with barriersqualitativeCanada, OntarioVorobyova et al., 2022 [35]Oct-22Aging/Elderly housing options, Stigma (HIV-related, sexuality, race), Housing-related HIV health outcomes/healthcare utilization, Sexuality, Social and community support, Other qualitativeCanadaMitchell et al., 2023 [36]Feb-23Aging/Elderly housing options, Stigma (HIV-related, sexuality, race), Housing-related HIV health outcomes/healthcare utilization, Sexuality, Social and community support, Other—health comorbidities, economic, food, and job insecurity, lack of transportationqualitative—mixed method designUSAWeinstein et al., 2023 [37]Mar-23Aging/Elderly housing options, Stigma (HIV-related, sexuality, race), Housing-related HIV health outcomes/healthcare utilization, Sexuality, Social and community support, Other—illicit substance use, and depression. financial hardship due to HIV-related disability, and social isolation related to decades of marriage inequality).quantitativeUSA, Florida

## 6. Quality of Included Studies and Risk of Bias Assessment

Peer-reviewed journal articles were the only ones considered and included in the study. The flow chart documented the review process to maintain the transparency, integrity, and creditability of the PRISMA systematic review’s [16,38]. Inter-rater reliability was not included because the experts who reviewed the files interacted and discussed the classifications until an agreement was reached on the terminology.

## 7. Latent Semantic Analysis (LSA)

Data for this analysis were text from 23 abstracts selected from the PRISMA review articles outlined above using SAS Enterprise Miner 15.2. Latent Semantic Analysis (LSA) is a theory and technique that uses statistical calculations on a sizable corpus of text to extract and represent the meaning of words used in context [39]. LSA complements the systematic review’s manual process by facilitating objectivity, transparency, and efficiency throughout the literature analysis phase. Its application, in conjunction with SLR, has been documented [40,41,42]. The fundamental tenet is that the total word contexts in which a particular word appears and does not appear establish a system of reciprocal limitations that predominantly dictate the degree of semantic similarity between individual words and word groups/sets. Topic extraction is a widely used method for efficiently analyzing extensive document collections and identifying common themes by identifying keywords within the documents. Applying SAS Text Miner default settings, Singular Value Decomposition (SVD) (low SVD resolution and maximum cluster) was used to cluster text and identify 15 descriptive terms that fully represent each cluster from the selected 23 abstracts (dataset). Our dataset’s observations are grouped using a process called clustering, which creates comparable observations within each group and dissimilar observations between them. Based on the existence of related topics, clustering separates the collection of documents into different groups in the context of text mining.

## 8. Discussion

This section shows the LSA term and Topic modeling results, Terms by document, and Topic by document reports. Table 1 shows the frequencies of the top 20 relevant terms, removing some search words in our PRISMA inclusion criteria (HIV/aids, old/age/adult/older adult, housing); important terms are care, health, live, service, treatment, social, barrier, stigma, and experience.

Applying SAS Text Miner default settings (low SVD resolution and maximum cluster) and using 15 descriptive terms that fully represent each cluster from the selected 23 abstracts from the systematic review, the analysis yielded two clusters.

Table 2 shows that the largest cluster (Cluster 1) has 70% of the documents relating primarily to older adults’ barriers to healthcare services and housing and the need for increased research and improvement in policies regarding overall health, well-being, and quality of life. The second cluster, with 30%, illustrates a critical support resource—social support for both emotional (mental health) and functional needs and uncertainties associated with finance, food, and sexuality insecurities. The two clusters have been named based on the main terms in each cluster, which represent the themes of the terms in those clusters. Cluster 1: Improvements to access housing and healthcare services and policies. Cluster 2: Unmet needs—social support, mental health, finance, food, and sexuality insecurities.

## 9. Topic Extraction

A topic represents a group of terms that describe a theme or an idea. Documents in this corpus (23 abstracts selected for analysis) can be assigned a score representing the degree of association for a topic, which might have zero, one, or several topics. Creating a topic list establishes different combinations of words of interest in the study articles. The Text Topic Node in SAS Enterprise Miner 15.2 was applied to reveal topics from a text. The node computes term topic weight and document topic weight. These are used to calculate cut-off scores for each multi-term Topic. Term cut-off represents the most decisive match to which a term belongs to a topic, and document cut-off is the most decisive match that determines if a document belongs to a topic. Table 3 illustrates the four topics extracted from our corpus.

Table 4 illustrates the four topic terms, and the formulated emerging key themes from our PRISMA-selected articles. The themes are as follows: Topic ID. 1/Theme: Holistic care approach elements, Topic ID. 2/Theme: Insecurities—Food, financial, sexuality, and other basic needs, Topic ID. 3/Theme: Access to housing and treatment/care, and Topic ID. 4/Theme: Homelessness and HIV-related outcomes. Table 5 shows how clusters relate to the Topic I.D.s; Cluster 1 encompasses Topic I.D.s 1, 3, and 4, while Cluster 2 has Topic ID 2.

The 23 articles selected in this systematic review span 11 years (2012–2023) and provide diverse research study themes. The clusters illustrate important social determinants of health and public health, healthcare interventions, and policies that are needed for HIV older adults. Table 6 shows a detailed representation of the recurrent themes. The top three frequently mentioned themes in the articles are as follows: Topic ID. 1/Theme: Holistic care approach elements (91%), Topic ID. 2/Theme: Insecurities—Food, financial, sexuality, and other basic needs (87%), and Topic ID. 4/Theme: Homelessness and HIV-related outcomes (83%). These three themes recurred consistently in recent research studies in the corpus published from 2021 to 2023.

## 10. Discussion

The emerging Topic ID. 1 shows that some documents reveal elements of holistic care and the need for a holistic person-centered care approach. The number of older people living with HIV (PWH) is increasing, and this presents a growing concern regarding their care or standard care model. Older adults with HIV exhibit comorbidities frequently linked to aging and, compared to HIV-negative individuals, have a higher risk of dementia, diabetes, frailty, depression, osteoporosis, and some malignancies [43]. People with HIV experience delays in treatment in general due to fear of disclosure, loneliness, social isolation, and depression brought on by the stigma attached to the virus, and they may also perceive discrimination from healthcare professionals, which makes them reluctant or unwilling to seek medical attention [44]. An ideal care model [45] identified three core care components: collaboration and integration, organization of geriatric care, and support for holistic care that may enhance the adequate provision of geriatric care to individuals living with HIV. A holistic person-centered care approach for older adults with HIV needs to incorporate financial, social, emotional/spiritual, and physiological aspects. Implementing this requires significant investments in healthcare and supportive services that are accessible and equitable.

Topic ID. 2 shows insecurities about finances, food, sexuality, and other basic needs. Due to low income from precarious wage jobs and federal disability benefits, older HIV individuals face financial insecurity, which leads to interconnected and overlapping vulnerabilities concerning housing, healthcare, and food [28]. These insecurities have a compound effect on the general well-being of older adults with HIV. Previous research provides evidence of the connection between older persons with HIV and their physical and mental health as well as their health-related quality of life (HRQoL) and unmet basic requirements (such as food, clothing, or stable housing). A high frequency of unmet requirements harms people’s health and quality of life [23]. Wainwright et. al. [29] emphasized the significance of improved service delivery, care, and treatment for older persons living with HIV who are homeless. Medicare-eligible PLWH in U.S. nursing homes do not qualify for HIV drug-reducing programs due to their status as profit-making institutions. Cost-sharing assistance programs are only available for low-income community-dwelling older adults, making anti-retroviral therapy drugs accessible but not for nursing homes, causing financial burdens [26]. Improvements in policies are needed to reduce financial barriers due to the cost of drugs in nursing homes, enabling access to these settings and consistent supply of essential antiretroviral treatment for residents.

Isolation, stigma, and ageism were prevalent at high rates in gay and bisexual men, classified as “the gay dilemma”; stigma was due to the following: (1) identification as gay or bisexual; (2) being HIV-positive; and (3) being an older individual. Concerns about stigma, provider competency, affordable housing, and financial stability were prevalent and observed in the subpopulation of sexual minorities [33].

Topic IDs 3 and 4 reveal challenges associated with homelessness, access to housing and treatment/care, and HIV-related care/health outcomes. While prior research raised concerns regarding HIV-infected older persons’ acceptance into retirement homes and long-term care facilities, ref. [29] identified barriers to subsidized housing based on their age, HIV status, and sexuality. On top of ageism and HIV-related stigma, older sexual minorities with HIV may encounter extra hurdles. They face housing insecurity because of identity-based stresses such as financial hardship and social isolation caused by decades of marriage inequity [17]. Ref. [33] identified similar psychosocial challenges affecting older adult sexual minorities, including housing and financial insecurity, provider competency, and stigma, with the subpopulation of gay and bisexual men experiencing isolation, stigma, and ageism. Older adults with HIV encounter challenges accessing social and other support services at home due to trauma, stigma, and uncertainty about the future [34]. Programs for housing assistance should be given top priority when developing public health policies since they have been demonstrated to improve HIV-related health outcomes and lower risk behaviors. Older HIV adults agonized over lacking a safe physical space for conversing and interacting with peers, which could potentially help in enhancing health maintenance, physical/mental well-being, and quality of life [21].

Formal (long-term and short-term care facilities) and informal (owned or rental/subsidized housing) housing options are available to older persons living with HIV. Older adults with HIV who experience homelessness or housing instabilities are more susceptible to other stresses, including trauma, financial insecurity, loneliness, and prejudice, which can result in marginalization and psychosocial vulnerability [46]. According to [30], loneliness is a crucial problem among older people living with HIV (PLWH). Forty percent of those who reported feeling lonely had previously experienced homelessness or unstable housing. These issues could have significantly impacted the living experiences of older PLWHA. There were contributing observations of ageism, sexism [18,33], and HIV-related stigma [17,19,22,23,26,28,29,33,37,41,42,43,44,45,47].

Levels of HIV-related stigma are elevated in nursing homes, which prevents older adults with HIV from entering long-term care institutions. Preference for home-based care was higher among older PWH, with self-care coming in second. Fewer people favor mutual aid, community-based, or institutional care [24,48]. Provider competency, staff members’ trust in HIV care, and readiness for the elderly HIV population should be enhanced through training to reduce barriers associated with stigma, discrimination, sexuality, and transgender subpopulations [15,17,25,33,36,48]. The presence of the terms barrier, stigma, and housing in our LSA top twenty terms in our corpus sheds insight and answers our research questions, in that stigma and discrimination levels (based on HIV status and sexuality) are prevalent and act as barriers to subsidized housing, long-term care, and HIV care facilities, and antiretroviral treatment adherence [15,17,19,22,35,36]. A structural barrier that impedes persistent viral suppression, adherence to anti-retroviral therapy, and access to vital HIV medical services (holistic care) is housing instability [49]. The implications for interventions include removing structural barriers to care at the individual (financial, food, and housing insecurities), community (stigma, discrimination, transportation logistics), and healthcare (stigma/discrimination, provider competency) levels. These interventions provide an opportunity for healthcare policymakers to achieve healthcare equity for the elderly living with HIV/AIDS. Clinicians and policymakers should address individual barriers, adopt a patient-centered approach, increase clinician visits, and provide competency training on sexual orientation and gender identity. They should also ensure long-term follow-up, involve families or caregivers, and improve patient education in care management. The implications of theory and insights will be beneficial in contributing to the formulation and knowledge of HIV/AIDS geriatric care models. These implications for practice and policy include recommendations for reducing structural barriers to care by enhancing patient confidence in the health delivery system and for aligning services toward compassionate and inclusive care. Future research is needed on evidence-based care models in the respective care preferences that provide better health outcomes and quality of life.

## 11. Strengths and Limitations

This PRISMA systematic review has several strengths. The search method robustly covers many databases to identify articles for the study. The PRISMA 2020 statement was followed to determine eligibility and quality assessment. Some restrictions should be considered as well. Studies from other industrialized nations were not considered for this study; only studies from the USA and Canada were included. We could have missed some insightful, pertinent, and educational articles based on empirical research reports inaccessible in the commercially published literature because our gray literature searches were limited. Although the text corpus of 23 articles may be regarded as small, it represents the current body of evidence that gives insight into this understudied area of housing experiences of older adults with HIV. This study can be reinvestigated in future research when more articles are published, but the current investigation of all 23 published works sets a baseline for future work.

## 12. Conclusions

Our study’s strength is combining a systematic review and a latent semantic analysis (LSA) to demonstrate and understand the relationships between the terms in the corpus that gives focus to the emerging topics/themes affecting older adults living with HIV. The review articles and LSA highlight themes of a holistic care approach, insecurities, access to housing and treatment, homelessness, and HIV-related outcomes. When these are clustered, two significant themes emerge (improve policy and housing access; unmet needs—social support, mental health, finance, food, and sexuality insecurities), which provide insights for future research and policy formulation to improve the health and quality of life of older adults with HIV. We recommend policies that implement a holistic care approach for this population to make sure some do not get lost in the system, significant investment in healthcare, and supportive services that are accessible and equitable. If prioritized, the two North American countries have the organizational, financial, professional, and cultural capacity to implement these recommendations. Future research should involve the following: (1) developing an instrument that measures the unmet basic needs of this population; (2) the effectiveness of care models and formulation of an accessible and equitable standard care model; (3) reduction of HIV-related stigma and strengthening social support for older adults living with HIV; (4) evidence-based care models that reflect the care preferences of older adults with HIV and deliver better health outcomes, well-being, and quality of life.

## Figures and Tables

**Table 1:** Top twenty terms.

Term ID	Term	Frequency	Number of Documents
1	HIV	94	23
2	live	27	19
3	old	51	18
4	health	34	16
5	care	60	14
6	adult	19	12
7	study	14	12
8	age	20	11
9	older adult	18	11
10	experience	12	10
11	year	13	10
12	research	13	10
13	social	18	10
14	stigma	15	10
15	age	16	9
16	barrier	17	9
17	experience	15	9
18	finding	10	9
19	house	13	9
20	participant	15	9

**Table 2:** Cluster names and Cluster Descriptive terms.

Cluster ID	Cluster Name	Cluster Description	Frequency	Percentage
1	Improvements to access to housing and healthcare services and policy formulation	+increase + associate + improve + policy + home + adult + ‘older adult’ + barrier care + service aids + explore research + finding + study	16	70%
2	Unmet needs—social support, mental health, finance, food, and sexuality insecurities.	+woman + conduct + man + theme mental + uncertainty basic financial food qualitative social + interview + provider approach + include	7	30%

**Table 3:** Topics.

Gategory	Topic ID	Document Cutoff	Term Cutoff	Topic	Number of Terms	Number of Documents
Multiple	1	0.001	0.13	+service, +service, treatment, +include, care	17	21
Multiple	2	0.001	0.13	+uncertainty, +woman, +man, food, basic	17	20
Multiple	3	0.001	0.129	PLWH, home, +home, art, access	10	15
Multiple	4	0.001	0.13	homelessness, HIV-related, +outcome, +associate, care	20	19

**Table 4:** Topics table from the Text Topic Node.

Topic ID	Topic	Theme	Number of Terms	Number of Documents
1	+service, +service, treatment, +include, care	Holistic Care Approach elements	17	21
2	+uncertainty, +woman, +man, food, basic	Insecurities—Food, financial, sexuality, and other basic needs	17	20
3	PLWH, home, +home, art, access	Access to housing and treatment/care	10	15
4	homelessness, HIV-related, +outcome, +associate, care	Homelessness and HIV-related health outcomes	20	19

**Table 5:** Cluster names and Topic ID: Descriptive terms.

Cluster ID	Cluster Name	Topic ID; Terms
1	Improvements to access to housing and healthcare services and policy formulation	Topic 1; +service, +service, treatment, +include, care Topic 3; PLWH, home, +home, art, access Topic 4; homelessness, HIV-related, +outcome, +associate, care
2	Unmet needs—social support, mental health, finance, food, and sexuality insecurities.	Topic 2; +uncertainty, +woman, +man, food, basic

**Table 6:** Systematic Review Articles Summary Topic/Thematic Analysis.

			Cluster 1			Cluster 2
			Improvements to access to housing and healthcare services and policy formulation			Unmet needs—social support, mental health, and finance and food insecurities.
#	Author	Date	Topic 1. +service, treatment, +include, care	Topic 3; PLWH, home, +home, art, access	Topic 4; homelessness, HIV-related, +outcome, +associate, care	Topic 2; +uncertainty, +woman, +man, food, basic
1	Furlotte et al., 2012 [15]	Mar-12	1	1	1	1
2	Lane et al., 2013 [14]	Jan-13	1	1	1	0
3	Solomon et al., 2014 [17]	Feb-14	1	0	1	1
4	Arnold et al., 2017 [18]	Jan-17	1	1	1	1
5	Cox and Brennan-Ing, 2017 [19]	Jan-17	1	1	1	1
6	Siou et al., 2017 [20]	May-17	1	1	1	0
7	Tobin et al., 2018 [21]	Jan-18	1	1	0	1
8	Solomon et al., 2018 [22]	Apr-18	1	1	1	1
9	Sok et al., 2018 [23]	May-18	0	0	1	1
10	Nguyen et al., 2019 [24]	Feb-19	1	0	1	1
11	Baguso et al., 2019 [25]	Apr-19	1	1	1	1
12	Olivieri-Mui, 2019 [26]	May-19	1	1	0	1
13	Justice and Akgün, 2019 [27]	Jul-19	1	1	1	1
14	Whittle et al., 2020 [28]	Jan-20	0	0	1	1
15	Wainwright et al., 2020 [29]	Jun-20	1	1	1	1
16	Yoo-Jeong et al., 2020 [30]	Jul-20	1	0	1	1
17	Chayama et al., 2020 [31]	Aug-20	1	1	0	0
18	Koehn et al., 2021 [32]	Jan-21	1	1	0	1
19	Cherry et al., 2021 [33]	Jun-21	1	0	1	1
20	Murzin et al., 2022 [34]	Sep-22	1	1	1	1
21	Vorobyova et al., 2022 [35]	Oct-22	1	0	1	1
22	Mitchell et al., 2023 [36]	Feb-23	1	0	1	1
23	Weinstein et al., 2023 [37]	Mar-23	1	1	1	1
	Total		21	15	19	20
	Frequency %		91	65	83	87

## Data Availability

Not applicable.

## Supplementary Materials

The following supporting information can be downloaded at: https://www.mdpi.com/article/10.3390/healthcare12100992/s1, PRISMA Checklist—Systematic Review: HIV, Aging, and Housing—North American Perspective 2012–2023.
