# Supplementary material for: Systematic Review: HIV, Aging, and Housing—A North American Perspective, 2012–2023
Source: Healthcare (Basel). 2024 May 11;12(10):992. doi: 10.3390/healthcare12100992 (PMC11121341; doi:10.3390/healthcare12100992)
Supplement: Supplementary file 1 [file healthcare-12-00992-s001.zip › healthcare-2957806-supplementary.pdf]

PRISMA Checklist_ Systematic Review: HIV, Aging, and Housing – North American Perspective 2012-2023

	Item #	Checklist item	Page location where item is reported
**TITLE**			
Title	1	**Systematic Review: HIV, Aging, and Housing - North American Perspective 2012-2023**	1
**ABSTRACT**			
Abstract	2	Advances in anti-retroviral therapy (ART) have decreased mortality rates and subsequently led to a rise in the number of HIV-positive people living longer. The housing experiences of this new population of interest - older adults (50 years and older) living with HIV are under-researched. Understanding the housing experiences and unmet needs of older HIV people can better provide comprehensive care services for them. This study's systematic review evaluated peer-reviewed literature reporting housing access/insecurity/assistance/options, housing impact, and unmet needs of older individuals living with HIV in North America from 2012 to 2023. Furthermore, Latent Semantic Analysis (LSA), a text-mining technique, and Singular Value Decomposition (SVD) for text clustering were utilized to examine unstructured data from the abstracts selected from the review. The goal allowed for a better understanding of the relationships between terms in the articles and the identification of emerging public health key themes affecting older adults living with HIV. The results of text clustering yielded two clusters focusing on 1). Improvements to housing and healthcare services access and policies. 2). Unmet needs - social support, mental health, finance, food, and sexuality insecurities. Topic modeling demonstrated four topics, which we themed to represent 1. Holistic Care Approach; 2. Insecurities - Food, financial, sexuality, and other basic needs; 3.Access to housing and treatment/care, and 4. Homelessness and HIV-related health outcomes. Stable housing, food, and healthcare services access and availability are critical elements to incorporating comprehensive, holistic healthcare for older adults living with HIV. This aging population needs high-priority policies that invest in equipping healthcare and supportive services that are accessible and equitable in the future.<br>**Keywords**: HIV, Older adults, Stigma, Sexuality, Housing, Homelessness.	1 & 2
**INTRODUCTION**			
Rationale	3	Prisma systematic review will be used to investigate the current knowledge of homelessness, housing access/insecurity/assistance/options, housing implications, and unmet needs of older people living with HIV/AIDS (PLWHA) in North America.	2-4
Objectives	4	My research questions will explore insights and themes that support knowledge synthesis from PRISMA to reflect the current evidence on social determinants of health and unfulfilled needs for older PLWHA. This study will aim to answer the questions: How does the stigma affect fair housing options (retirement/assisted living/other forms of housing)? How does sexuality correlate with housing access and maintenance? How important is housing as a health determinant for older PLWHA? Is the HIV/AIDS stigma and discrimination still an issue for older PLWHA?	2-4
**METHODS**			
Eligibility criteria	5	**Inclusion Criteria:** Date of publication - (January 2012 – 2023, 11 years), HIV diagnosis, the Language of publication – English, translation to English availability, type of articles includes full text/scholarly and peer-reviewed, study methodology – quantitative, qualitative (Interviews, focus groups, ethnography), mixed-method with at housing status and HIV/AIDS health outcome as a variable. Geographic location (s) - North America (USA and Canada), Age of subjects >50yrs, Socioeconomic level (low SES)<br>**Exclusion Criteria:** Age of subjects <18yrs/ <50/65yrs, HIV status- negative, Socioeconomic level (High SES), Country – Not USA or Canada, Full articles not available, Language – not English/Translation not available, Date of publication - (>11yrs), Type of publication – letters, editorials, non-peer-reviewed articles, dissertation/thesis, news and conference articles.	5-6
Information sources	6	A search was conducted using EBSCO Host databases, and most articles were retrieved from MEDLINE, Academic Search Complete, APA PsycINFO, Health Source: Nursing/Academic Edition, and Psychology and Behavioral Sciences Collection. Last searched 4/2023.	5
Search strategy	7	The Boolean phrase search criteria include the terms HIV or aids or acquired human immunodeficiency syndrome or human immunodeficiency	5

	Item #	Checklist item	Page location where item is reported
		virus AND elderly or aged or older or elder or geriatric AND housing or (retirement home) or (nursing homes) or (care homes) or (long-term care) or (residential care) or (aged care facility). Peer-reviewed articles from 2012 to 4/2023	
Selection process	8	Study selection was followed the process of identification (3172 articles), screening (1743 articles), eligibility (66 articles), and inclusion (23 articles).<br>An initial screening involved removing duplicates and based on title and abstract we excluded irrelevant items from the comprehensive search, as 'false positives' are inevitable when designing a search for comprehensiveness rather than precision. We sought full-text 133 articles, more articles were excluded because no issues involving HIV, housing, no full text, and no English translation.<br>Two reviewers independently screened each record and each report. No automation tools were used for this process.	6-7
Data collection process	9	Study selection was followed the process of identification (3172 articles), screening (1743 articles), eligibility (66 articles), and inclusion (23 articles).<br>An initial screening involved removing duplicates and based on title and abstract we excluded irrelevant items from the comprehensive search, as 'false positives' are inevitable when designing a search for comprehensiveness rather than precision. We sought full-text 133 articles, more articles were excluded because no issues involving HIV, housing, no full text, and no English translation.<br>Two reviewers independently screened each record and each report. No automation tools were used for this process.	6-7
Data items	10a	The outcomes include older adults' barriers to healthcare services and housing, critical support resources such as social support for both emotional (mental health) and functional needs, uncertainties associated with finance, food, and sexuality insecurities, and other unmet needs. The outcomes were compatible with each study and used SAS Enterprise Miner 15.2. Latent Semantic Analysis (LSA).	8-15
	10b	No other data was sought, and no assumptions were made about any missing or unclear information.	n/a
Study risk of bias assessment	11	Studies from other industrialized nations were not considered for this study; only studies from the USA and Canada were. We could have missed some insightful, pertinent, and educational articles based on empirical research reports inaccessible in commercially published literature because our gray literature searches were limited.<br>Two reviewers independently assessed each study and no automation tools used.	16
Effect measures	12	No effect measure was computed.	n/a
Synthesis methods	13a	Latent Semantic Analysis (LSA) is a theory and technique that uses statistical calculations on a sizable corpus of text to extract and represent the meaning of words used in context. Topic extraction using LSA is used to efficiently analyze extensive study and identify common themes by identifying keywords within them. Also Singular Value Decomposition (SVD) (low SVD resolution and maximum cluster) was applied to cluster text and using 15 descriptive terms that fully represent each cluster from the selected 23 abstracts (dataset) from the systematic review.	8-12
	13b	A summative content analysis was used for data presentation or synthesis.	12
	13c	A summative content analysis was used for data presentation or synthesis. Ones and zeros were put on the table to represent if the article discussed the title topics that emerged from the LSA. Percentage for each topic was calculated.	12
	13d	No meta-analysis was performed	n/a
	13e	No methods were used to explore possible causes of heterogeneity among study results (e.g., subgroup analysis, meta-regression).	n/a

	Item #	Checklist item	Page location where item is reported
	13f	No sensitivity analyses conducted to assess robustness of the synthesized results.	n/a
Reporting bias assessment	14	No methods were used to assess risk of bias	n/a
Certainty assessment	15	No methods are used to assess certainty (or confidence) in the body of evidence for an outcome.	n/a
**RESULTS**			
Study selection	16a	Study selection was followed the process of identification (3172 articles), screening (1743 articles), eligibility (66 articles), and inclusion (23 articles).<br>An initial screening involved removing duplicates and based on title and abstract we excluded irrelevant items from the comprehensive search, as 'false positives' are inevitable when designing a search for comprehensiveness rather than precision. We sought full-text 133 articles, more articles were excluded because no issues involving HIV, housing, no full text, and no English translation.<br>Two reviewers independently screened each record and each report. No automation tools were used for this process.	6-7

Prisma Systematic Review flowchart

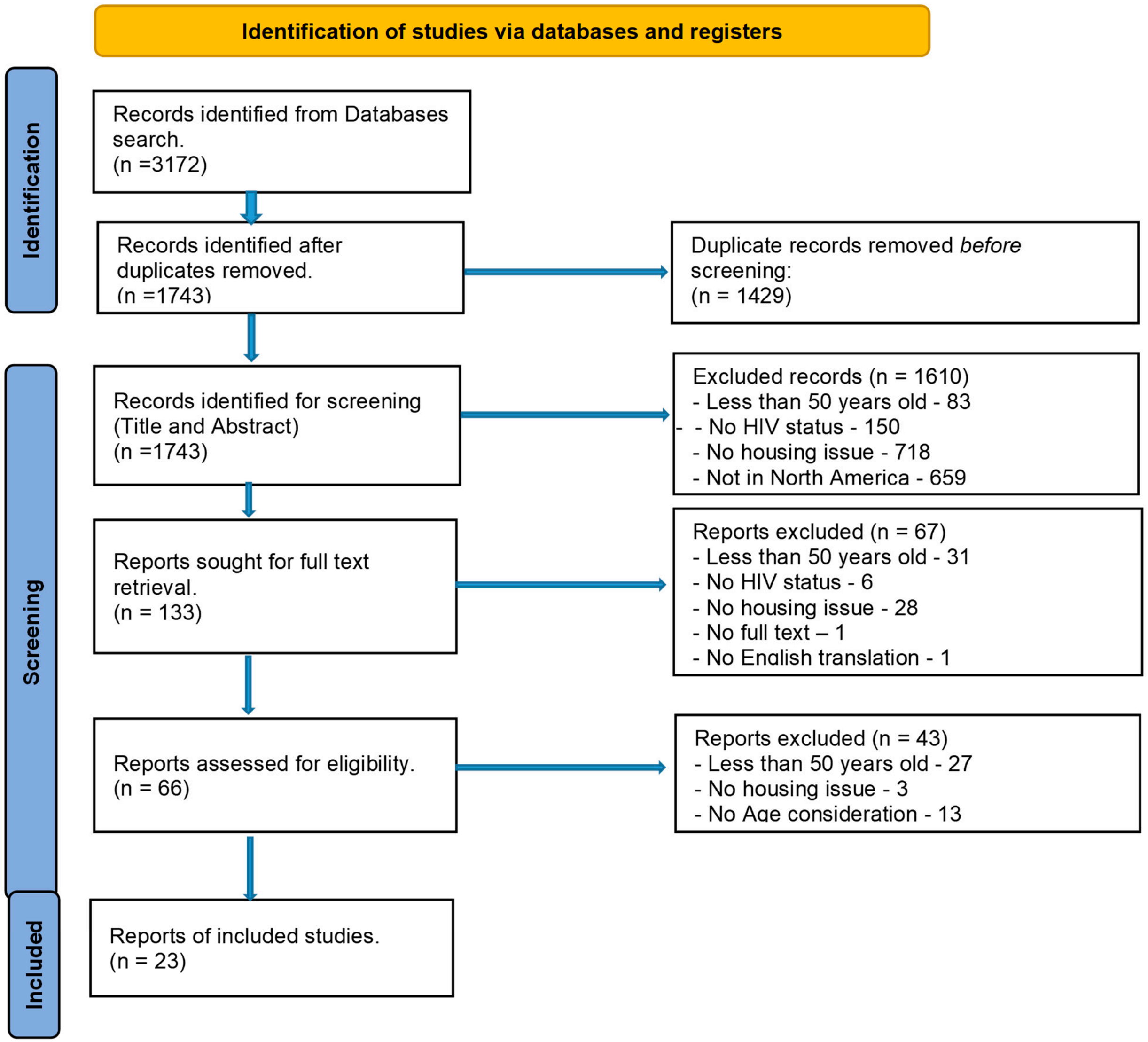

Results

	Item #	Checklist item	Page location where item is reported
	16b	Studies that might appear to meet the inclusion criteria were excluded because they did not have a specific age differentiation.	6-7
Study characteristics	17	Cite each included study and present its characteristics.	12
Risk of bias in studies	18	n/a	n/a
Results of	19	See step 17	12

				CLUSTER 1		CLUSTER 2
			Improvements to access to housing and healthcare services and policy formulation			Unmet needs - social support, mental health, and finance and food insecurities.
#	Author	Date	TOPIC 1.+service, treatment,+include,care	TOPIC 3; plwh,home, +home,art, access	TOPIC 4; homelessness, hiv-related, +outcome,+associate,care	TOPIC 2; +uncertainty, +woman,+man,food,basic
1	Furlotte et al., 2012	Mar-12	1	1	1	1
2	Lane et al., 2013	Jan-13	1	1	1	0
3	Solomon et al, 2014	Feb-14	1	0	1	1
4	Arnold et al., 2017	Jan-17	1	1	1	1
5	Cox and Brennan-Ing, 2017	Jan-17	1	1	1	1
6	Siou et al., 2017	May-17	1	1	1	0
7	Tobin et al., 2018	Jan-18	1	1	0	1
8	Solomon et al, 2018	Apr-18	1	1	1	1
9	Sok et al., 2018	May-18	0	0	1	1
10	Nguyen et al., 2019	Feb-19	1	0	1	1
11	Baguso et al., 2019	Apr-19	1	1	1	1
12	Olivieri-Mui, 2019	May-19	1	1	0	1
13	Justice and Akgün, 2019	Jul-19	1	1	1	1
14	Whittle et al., 2020	Jan-20	0	0	1	1
15	Wainwright et al., 2020	Jun-20	1	1	1	1
16	Yoo-Jeong et al., 2020	Jul-20	1	0	1	1
17	Chayama et al., 2020	Aug-20	1	1	0	0
18	Koehn et al., 2021	Jan-21	1	1	0	1
19	Cherry et al., 2021	Jun-21	1	0	1	1
20	Murzin et al., 2022	Sep-22	1	1	1	1
21	Vorobyova et al., 2022	Oct-22	1	0	1	1
22	Mitchell et al., 2023	Feb-23	1	0	1	1
23	Weinstein et al., 2023	Mar-23	1	1	1	1
	Total		21	15	19	20
	Frequency %		91	65	83	87

	Item #	Checklist item	Page location where item is reported
individual studies			
Results of syntheses	20a	See step 17	12
	20b	n/a	n/a
	20c	n/a	n/a
	20d	n/a	n/a
Reporting biases	21	n/a	n/a
Certainty of evidence	22	n/a	n/a
**DISCUSSION**			
Discussion	23a	. The themes were identified and named based on LSA topic extraction, Topic ID. 1/Theme: Holistic care approach elements, Topic ID. 2/Theme: Insecurities – Food, financial, sexuality, and other basic needs, Topic ID/Theme; Access to housing and treatment/care, and Topic. 4/Theme: Homelessness and HIV-related outcomes. The two clusters were identified and named based on each cluster's main terms, representing the themes of the terms in those clusters. Cluster 1: Improvements to access housing and healthcare services and policies. Cluster 2: Unmet needs - social support, mental health, finance, food, and sexuality insecurities. These findings in our review had supporting articles within and outside our selected articles.	8
	23b	Only studies from the USA and Canada were selected; no study from other developed countries was considered. Because our gray literature searches were limited, we could have missed some insightful, pertinent, and educational articles based on empirical research reports inaccessible in commercially published literature.	16
	23c	These may include risks of bias, such as selection bias. This study limited this by using and comparing review from independent reviewers.	6-7
	23d	The study uses a systematic review and latent semantic analysis to identify emerging themes affecting older adults living with HIV. It focuses on holistic care approach, insecurities, housing and treatment access, homelessness, and HIV-related outcomes. Two significant themes emerge: improving policy and housing access and addressing unmet needs like social support, mental health, finance, food, and sexuality insecurities. The study recommends policies implementing a holistic care approach, significant healthcare investment, and accessible, equitable supportive services. Future research should focus on developing an instrument to measure unmet basic needs, reducing HIV-related stigma, and developing evidence-based care models that reflect care preferences for older adult	16-17
**OTHER INFORMATION**			
Registration and protocol	24a	The review was not registered.	n/a
	24b	A protocol review was not prepared.	n/a
	24c	Describe and explain any amendments to information provided at registration or in the protocol.	n/a
Support	25	No financial or non-financial support for the review	n/a
Competing interests	26	The review authors have no competing interests.	n/a

PRISMA Checklist_ Systematic Review: HIV, Aging, and Housing – North American Perspective 2012-2023

	Item #	Checklist item	Page location where item is reported
Availability of data, code and other materials	27	All resources used for this study are publicly available.	n/a

From: Page MJ, McKenzie JE, Bossuyt PM, Boutron I, Hoffmann TC, Mulrow CD, et al. The PRISMA 2020 statement: an updated guideline for reporting systematic reviews. BMJ 2021;372:n71. doi: 10.1136/bmj.n71

For more information, visit: http://www.prisma-statement.org/

Statement

This study used a systematic review (PRISMA) to provide a detailed, transparent investigation of previous research on the current knowledge of homelessness, housing access/insecurity/assistance/options, and the implications of housing on older PLWHA in North America. The process was documented as illustrated in the flow chart in maintaining transparency, integrity, and credibility of the PRISMA systematic review PRISMA systematic review (Page et al., 2021).
